# Endothelial Progenitor Cells in Diabetic Microvascular Complications: Friends or Foes?

**DOI:** 10.1155/2016/1803989

**Published:** 2016-05-29

**Authors:** Cai-Guo Yu, Ning Zhang, Sha-Sha Yuan, Yan Ma, Long-Yan Yang, Ying-Mei Feng, Dong Zhao

**Affiliations:** ^1^Beijing Key Laboratory of Diabetes Prevention and Research, Luhe Hospital, Capital Medical University, Beijing 101149, China; ^2^Department of Endocrinology, Luhe Hospital, Capital Medical University, Beijing 101149, China

## Abstract

Despite being featured as metabolic disorder, diabetic patients are largely affected by hyperglycemia-induced vascular abnormality. Accumulated evidence has confirmed the beneficial effect of endothelial progenitor cells (EPCs) in coronary heart disease. However, antivascular endothelial growth factor (anti-VEGF) treatment is the main therapy for diabetic retinopathy and nephropathy, indicating the uncertain role of EPCs in the pathogenesis of diabetic microvascular disease. In this review, we first illustrate how hyperglycemia induces metabolic and epigenetic changes in EPCs, which exerts deleterious impact on their number and function. We then discuss how abnormal angiogenesis develops in eyes and kidneys under diabetes condition, focusing on “VEGF uncoupling with nitric oxide” and “competitive angiopoietin 1/angiopoietin 2” mechanisms that are shared in both organs. Next, we dissect the nature of EPCs in diabetic microvascular complications. After we overview the current EPCs-related strategies, we point out new EPCs-associated options for future exploration. Ultimately, we hope that this review would uncover the mysterious nature of EPCs in diabetic microvascular disease for therapeutics.

## 1. Introduction

*Prelude*. Diabetes is a type of metabolic disorder, featured as insulin resistance and insufficient insulin release due to pancreatic *β* cell dysfunction. Hyperglycemia appears in the early stage of diabetes. As the disease progresses, patients display excess thirst (polydipsia), frequent urination (polyuria), increased hunger (polyphagia), and loss of body weight. As most pathological changes involved in blood vessels of multiple organs, macro- and microvascular complications are frequently observed in diabetic patients and become the major cause of mortality.

Endothelial progenitor cells (EPCs) were first described nearly two decades ago. They participate in endothelial repair either by secreting angiogenic factors or by incorporating into disrupted endothelium and differentiating into endothelial cells to maintain endothelium integrity. Despite the long-term debate on the nature and identification of EPCs, compelling data showed that EPCs improved blood perfusion in peripheral ischemia. Nevertheless, abnormal angiogenesis is the featured pathological hallmark in diabetic retinopathy and nephropathy and, therefore, anti-VEGF treatment has been applied for treating the microvascular abnormality. Thus, the questions are rising: what is the nature of EPCs in diabetic microvascular disease? Could we apply EPCs for the treatment of diabetic retinopathy and nephropathy?

*The Presence of EPCs in Nature*. EPCs were first described in 1997. When CD34^+^ cells were isolated from human peripheral blood, they could differentiate into endothelial cells* in vitro* and participate in angiogenesis* in vivo* [1]. Despite a long debate about EPC identity, more and more data collectively indicated the presence of EPCs in nature: (1) human induced pluripotent cells (hiPSCs) could differentiate into vascular endothelial progenitors that could incorporate into injured endothelium* in vivo* [2, 3]; (2) despite being putative, both adult and human embryonic stem cells-derived hemangioblasts have shown endothelial capacities [4]; (3) different mechanical cues could sense cardiosphere-derived cells with enriched cKit^+^ subpopulation to differentiate either to endothelial or to cardiomyogenic lineage [5]; (4) Prox-1^+^ cells emerging at E9.5 could sprout from the veins to form lymph sacs and an initial lymphatic vasculature [6].

*Heterogeneous EPCs Population*. Data from different groups consistently demonstrated that EPCs are heterogeneous populations and classified into early EPCs and late EPCs. Early EPCs, also known as colony-forming unit-ECs (CFU-ECs) or CFU-Hill, exhibited a spindle shaped morphology, had poor proliferative capacity, and produced to a high extent angiogenic cytokines [1, 7]. By contrast, late EPCs, now generally termed as endothelial colony-forming cells (ECFCs), showed a cobblestone-shaped morphology and highly proliferative activity when cultured* in vitro*. In response to injury, they could be mobilized from bone marrow or other locations, migrate toward lesion site, and incorporate into injured endothelium* in vivo* [8–12]. Therefore, ECFCs are the main target under investigation. 

*Identification and Cultivation of EPCs*. So far, no unique surface marker has been identified for EPCs. Instead, different combinations of surface markers have been used for EPCs identification, such as CD34^+^/VEGFR2^+^ and CD133^+^/VE-cadherin^+^ [13–16]. Although these markers could help us to quantify EPCs* in vivo*, it is yet uncertain whether the cells isolated from these markers could give rise to ECFCs* in vitro*. Thus, functional assays become more reliable for EPCs confirmation, which include their morphology, ability to form vasculature* in vitro*, and incorporation into vasculature upon injection* in vivo*.

With regard to cultivation, early EPCs could be obtained from mononuclear cells (MNCs) of human peripheral blood or cord blood after 4-5 days of culture in medium containing endothelial growth factors and fetal calf serum [7, 17]. They are recognized by monocytic morphology, uptake of acetylated low-density lipoprotein (AcLDL), and binding to lectin.

To obtain ECFCs, mononuclear cells are cultured and passaged in endothelial cell specific medium with endothelial growth factors for at least 28 days. During culture, endothelial colonies appear and can be picked up for further expansion [16]. As the development of stem cell technology, embryonic stem cells and induced pluripotent stem cells (iPSCs) have become useful alternative cell sources to generate EPCs for practice [2, 3, 18, 19].

*Reduced Number and Impaired Function of EPCs in Diabetes*. Besides their huge capacity for endothelium generation and maintenance of endothelium integrity, a recent observation indicated the beneficial effect of EPCs on *β* cell survival. When pancreatic *β* cells were cotransplanted with EPCs, a better *β* cell engraftment with preserved function was observed, resulting in improved cure rate and initial glycemic control [20]. Unfortunately, EPCs number was significantly reduced with impaired function in diabetic patients as well as db/db mice, which was associated with poor vascular outcome in diabetes [21, 22]. In the next section, we discuss how hyperglycemia induces metabolic and epigenetic changes in EPCs.

## 2. Metabolic and Epigenetic Change of EPCs in Diabetes

### 2.1. Hyperglycemia-Associated Metabolic Change

Hyperglycemia induces advanced glycated end products (AGEs) formation and oxidative stress and increases reactive oxygen species (ROS) production in mitochondrion, which are the main killers of EPCs apoptosis [23]. Increased ROS production could also stimulate AGEs production, which further triggers ROS production. To make it worse, both of them synergistically activate nuclear factor-kappa B (NF-*κ*B) transcription [24]. NF-*κ*B is a well-known transcription factor with most of its target genes encoding inflammatory proteins inducing interleukin 1*β* (IL-1*β*) and tumor necrosis factor-*α* (TNF-*α*) and p53 as well as inducible nitric oxide synthase (iNOS). Thus, a loop between iNOS, ROS, NF-*κ*B, and AGEs develops to aggravate inflammation cascade [25–27]. Apart from ROS production in mitochondrion, high concentration of glucose provokes endoplasmic reticulum stress (ER stress), which promotes EPCs apoptosis and reduces EPCs migratory function [28]. Autophagy is a homeostatic process and is involved in organelle recycling and protein degradation. However, in response to high glucose-induced ER stress and oxidative stress, excessive autophagy might contribute to EPCs death [29].

**Figure 1:**
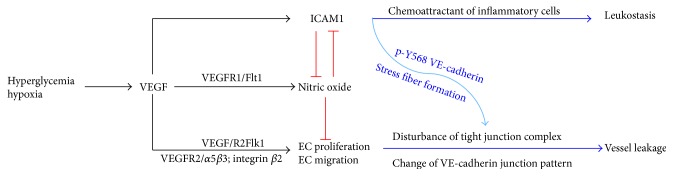
Uncoupling VEGF with nitric oxide in microvascular endothelial cells. Hyperglycemia promoted local VEGF production. VEGF stimulated endothelial cell proliferation via binding to VEGFR2. It induced endothelial cell migration and altered junction complex, in particular, VE-cadherin on the membrane. In parallel, VEGF promoted ICAM1 expression in endothelial cells which triggered inflammatory response to attack endothelium and made VE-cadherin dissemble. ICAM1 could also enhance ROS production that has negative impact on nitric oxide level and bioavailability. Although VEGF promoted nitric oxide production in endothelial cells via its receptor Flt1, nitric oxide is significantly reduced by ROS and oxidative stress, losing its inhibitory effect on VEGF-induced endothelial cell proliferation, migration, and activation.

In line with increased apoptosis by hyperglycemia, high level of glucose jeopardizes EPCs proliferative capacity partially via its inhibition of Akt phosphorylation and subsequently NOS activation or via activation of C-Jun N-terminal kinase (JNK) pathway [30–32]. High glucose also induces EPCs senescence by one of NF-*κ*B target genes, p53, and activation of p38MAPK pathway [33].  Figure 1 summarizes the molecular mechanisms of how hyperglycemia adversely affected EPCs number and function.

### 2.2. Hyperglycemia-Associated Epigenetic Change

Glycemic control is the initial treatment for diabetes. Nonetheless, tight glycemic control in the late stage of the disease fails to attenuate diabetic vascular complications. This phenomenon of glycemic memory was noticed in 1987 [34] and was extensively validated by different diabetic animal models and preclinical findings. These reports consistently pointed out that intensive glycemic control in the early stage could delay the development of diabetic microvascular abnormalities in type-1 [35] and type-2 diabetic subjects [36, 37] as well as streptozotocin-induced diabetic rats [38].

Glycemic memory is defined as the inexorable progression of diabetic vascular complication that is linked to uncontrolled hyperglycemia in the early stage of diabetes despite a tight glycemic control in the follow-on period. How does glycemic memory come?* In vitro* data have demonstrated that exposure of aortic endothelial cells to high glucose for 16 hours promotes NF-*κ*B p65 gene transcription. This transcription activation is sustained even when endothelial cells are cultivated back to normal glucose concentration [39]. Similarly, when Zheng et al. challenged retinal endothelial cells with high glucose conditions for one week and then returned to normal glucose conditions for two weeks, they noticed that one week of hyperglycemia was sufficient to induce NF-*κ*B activation which remained unchanged for the remaining two weeks [40]. They further found that Sirtuin 1 (SIRT1) was the main regulator in the event. Belonging to a class 3 HDAC, SIRT1 deacetylates H3K14 and H14K16 to control ROS production in endothelial cells and positively regulates EPCs differentiation into endothelial lineage [41].

Epigenetic modulation mainly includes posttranslational histone modification, DNA methylation, and microRNA-regulated transcriptional changes. As mentioned above, hyperglycemia-induced oxidative stress, ROS, and AGEs are the main factors for EPCs apoptosis and dysfunction. They are also potent inducers for epigenetic changes in EPCs. For instance, ROS is associated with a series of histone modifications in the promoter and enhancer of superoxide dismutase 2 (SOD2) gene in rat retinal endothelial cells [42]. High glucose triggers the increase of H3K4mel but decreases H3K9me2 and H3K9me3 expression level at the promoter of NF-*κ*B in human microvascular endothelial cells, leading to NF-*κ*B activation [43]. Furthermore, the histone codes H3K9ac, H3K12ac, H3K4me2, and H3K4me3 suppress eNOS transcription, leading to decreased nitric oxide [44]. All these epigenetic modifications accelerate proinflammatory machinery for EPCs apoptosis and loss of function.

It is well accepted that EPCs-mediated endothelium repair is beneficial for coronary heart disease, whereas abnormal angiogenesis is the hallmark of diabetic retinopathy and nephropathy and anti-VEGF agents have been applied for treating diabetic microvascular diseases. Thus, the question is coming: how do EPCs participate in the microvascular disease? Next, we first review how abnormal angiogenesis initiates diabetic microvascular disease.

## 3. Abnormal Angiogenesis in Diabetic Retinopathy and Nephropathy

Diabetic retinopathy is one of most frequent complications in diabetes, which is the leading cause of vision loss. After 20 years of diabetes, almost all type-1 diabetes patients, 80% of insulin-dependent diabetics, and 50% of insulin-independent type-2 diabetic patients will develop retinopathy [45, 46]. Diabetic retinopathy is traditionally classified into two main clinical forms: nonproliferative diabetic retinopathy (NPDR) and proliferative diabetic retinopathy (PDR), based on the presence or absence of neovascularization.

In a similar situation to diabetic nephropathy, 20%–40% of diabetic patients develop nephropathy [47], which is featured as initial microalbuminuria and then followed by a lot of albuminuria and increased serum creatinine level. Diabetic nephropathy has become the leading cause of end stage renal disease worldwide.

From the pathological view of diabetic retinopathy and nephropathy, aberrant angiogenesis is the common feature in the diseases, which is characterized as hypoxia-induced local VEGF expression, reducing nitric oxide level and availability, oxidative stress, vascular leakage, and inflammation. In parallel with VEGF axis, the imbalanced expression between angiotensin I and angiotensin II serves as another mechanism for endothelial dysfunction and hyperpermeability in diabetic eyes and kidneys.

### 3.1. Uncoupling VEGF with Nitric Oxide

Endothelium acts as physiological barrier between serum proteins and blood vessel, whose integrity is tightly controlled by nitric oxide produced by endothelial cells. Physical level of VEGF promotes endothelial cell proliferation via its receptor VEGFR2 and stimulates eNOS activation for nitric oxide (NO) production via VEGFR1.

In the setting of diabetes, hypoxia-induced VEGF production acts on endothelial cells for proliferation, migration, and NO production; in the meantime, it stimulates intercellular cell adhesion molecule-1 (ICAM1) expression on the surface of endothelial cells [48, 49]. ICAM1 triggers NADPH oxidase activation for reactive oxygen species (ROS) production [48]. ROS, advanced glycation end products (AGEs), asymmetric dimethylarginine (ADMA), and hyperglycemia dramatically increase arginase activity [50]. Arginase competes with NOS for the common substrate, L-arginine, resulting in insufficient substrate for NOS. Uncoupled NOS in turn use more oxygen molecules to generate superoxide, which catabolizes any available NO for peroxynitrite formation [50]. In addition, ADMA is an endogenous inhibitor of eNOS but its level is increased in diabetes [51]. Ultimately, NO level and availability are severely reduced.

In contrast to diminished NO availability, VEGF induces abnormal endothelial cell proliferation via VEGFR2 and, more importantly, enhances migration but disrupts cytoskeleton rearrangement through cross talk between VEGFR2 and integrin *α*5*β*3 or integrin *β*2, separately [52, 53]. Moreover, under physical angiogenesis, Notch/VEGFR2 modulates differential dynamics of VE-cadherin junction pattern during sprouting. When switched to pathological high VEGF condition, the differential VE-cadherin mobility is lost and thus tip cell competition and stalk cell intercalation are disturbed [54]. In parallel, downstream of VEGF-induced ICAM1 expression and ROS production, Src kinase and protein tyrosine kinase 2*β* are activated, both of which phosphorylate Y-658 on VE-cadherin for dissembling this protein [55]. Moreover, ICAM1 could also activate Rho GTPase for stress fiber formation, leading to permeability [55]. Nitric oxide antagonizes endothelial cell proliferation and inflammation, thereby maintaining endothelium integrity [56].

The pathological pattern “VEGF uncoupling with NO” is preserved and serves as the main mechanism in diabetic retinopathy and nephrology [52, 57]. For instance, studies from diabetic eNOS knockout mice have demonstrated that this mouse model develops severe albuminuria as well as increased VEGF expression in the kidney. Histological analysis confirms diabetic nephropathy in this model as evidenced by mesangial expansion, glomerular basement membrane thickening, and mesangiolysis [57]. Concerning the eye, VEGF level has been found to be significantly increased in ocular tissues in diabetic patients with retinopathy, which is accompanied with inflammation and uncoupled eNOS [58, 59].

**Figure 2:**
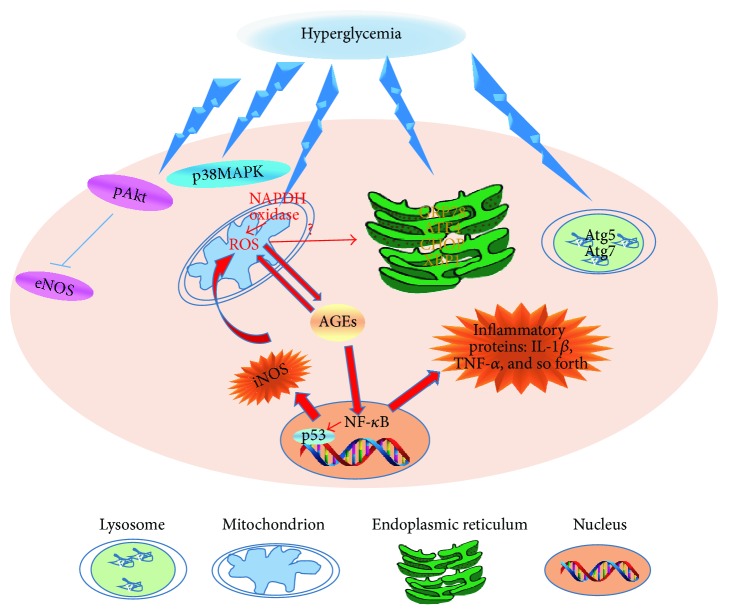
The detrimental effect of hyperglycemia on endothelial progenitor cells (EPCs) number and function. High level of glucose stimulates ROS production through activation of NAPDH oxidase. Increased reactive oxygen species (ROS) production triggers advanced glycated end products (AGEs) formation. AGE in turn further increases ROS production and, in the meantime, promotes nuclear factor-kappa B (NF-*κ*B) transcription. NF-*κ*B is crucially involved in inflammation via transcriptional activation of its target genes such as IL-1*β* and TNF-*α*. In parallel, NF-*κ*B also activates p53 to accelerate cell senescence and iNOS that further potentiates ROS production. Except mitochondrial diabetic retinopathy ion damage, hyperglycemia induces endoplasmic reticulum (ER) stress and excessive autophagy to further facilitate EPCs death. Apart from that, hyperglycemia inhibits Akt phosphorylation and subsequently eNOS activation. It also activates p38MAPK pathway to promote cell death and senescence. Ultimately, EPCs are triggered to undergo apoptosis and become dysfunctional.

Taken together, irregular endothelial proliferation, migration, cytoskeleton rearrangement, and dissembled VE-cadherin contribute to vessel leakage. The “uncoupling of VEGF with NO” pattern is shown in Figure 2.

### 3.2. Angiopoietins in Diabetic Eye and Kidney

On top of “VEGF uncoupling with NO,” angiopoietin 1 (Ang1)/Tie2 is another system that protects endothelium integrity. Upon binding to Tie2 tyrosine kinase receptor, Ang1 has been shown to reduce endothelium permeability, suppress NF-*κ*B-associated inflammation, and antagonize VEGF functions. In contrast, Ang2 is the endogenous antagonist of Ang1. The increase of Ang2 in diabetes condition competes with Ang1 for binding to Tie2, rendering the Ang1-regulated antiangiogenesis toward Ang2-mediated abnormal angiogenesis [60]. Recent study reported that VEGF and Ang1 exert opposing effect on endothelial cell permeability via their distinct modulation of RhoA-specific guanine nucleotide exchange factor (Syx). In the study, the authors elaborately showed that Syx was recruited to endothelial junction by Mupp1 and formed a complex with multiple members of the apicobasal polarity complexes (CRB) on the membrane for junction stabilization. They further demonstrated that VEGF caused Syx dissociation from Mupp1 and Syx translocation from cell junctions, resulting in junction disassembly [61].

A potent Ang1 variant, cartilage oligomeric matrix protein (COMP), was developed nearly one decade ago. Administration of COMP-Ang1 reverses hyperglycemia-induced kidney dysfunction by suppression of ICAM1 and monocyte chemoattractant protein-1 and monocyte/macrophage infiltration in diabetic db/db mice [60]. COMP-Ang1 also reduces renal tissue levels of transforming growth factor-beta1 (TGF-*β*1), alpha-smooth muscle actin, and fibronectin, as well as Smad 2/3 expression, but increases Smad 7 expression [60]. Likewise, recent data demonstrate that COMP-Ang1 could ameliorate retinopathy and stabilize blood retinal barrier in diabetic Ins2Akita mice [63].

## 4. EPCs in Diabetic Retinopathy and Nephropathy

### 4.1. EPCs in Diabetic Retinopathy

After observing the presence of abnormal angiogenesis in the development of diabetic retinopathy, antiangiogenesis therapies such as anti-VEGF agents have been taken to treat diabetic patients with retinopathy. This has brought a long debate: are EPCs good or bad in the disease? There is no definite answer so far. As the complicated pathogenesis and different types of diabetic retinopathy, either decreased or increased or unchanged EPCs number has been reported in diabetic patients with severe retinopathy when comparing to diabetic patients with no to mild retinopathy or healthy subjects [64–68].

To be noted, in the studies where they found increased EPCs number in the patients with diabetic retinopathy, EPCs function such as migration and mobilization and homing was often impaired. And this EPCs pattern, that is, increased EPCs number with impaired function, is consistently conserved in both type-1 and type-2 diabetic patients [68, 69]. Paradoxically, intravitreal delivery of COMP-Ang1 improves endothelial integrity and ameliorates vascular leakage by promoting the incorporation of endothelial colony-forming cells into retinal vasculature [63] in diabetic mice. Giving the nature of ECFCs in endothelial regeneration and NO production, this is an excellent example illustrating using the right ECFCs in reversing diabetic retinopathy.

### 4.2. EPCs in Diabetic Nephropathy

The early pathological features of diabetic nephropathy include hyperperfusion and hyperfiltration due to endothelial cell damage and abnormal angiogenesis. As the inflammation becomes more severe, glomeruli fibrosis develops, resulting in kidney failure. Although the exact mechanisms of nephropathy are not fully understood, AGEs, oxidative stress, and the activation of the renin-angiotensin-aldosterone system (RAAS) facilitate and strengthen these changes partially through activation of TGF-*β*1 signaling and increased vascular endothelial growth factor (VEGF) expression in the kidney toward progression of fibrosis and renal failure [70–72]. Therefore, antagonism of VEGF signaling using anti-VEGF antibody or endogenous inhibitor of VEGF or inhibition of VEGF receptor-1 phosphorylation has been used to improve early renal function in diabetic rats injected with STZ or db/db mice [73–75]. In parallel, inhibition of AGEs suppresses TGF-*β*1 and VEGF signaling pathways and alleviated diabetic nephropathy [72].

As endothelial cell damage occurs in the early stage of kidney dysfunction, its repair is not well processed due to EPCs defect in diabetes. In line with this, Makino and coworkers reported the negative correlation between EPCs number and microalbuminuria or albumin excretion rate in both type-1 and type-2 diabetic patients, respectively [76, 77], suggesting the protective effect of EPCs in the structure and function of glomeruli.

Putting these evidences together, ECFCs could be a promising target for treating diabetic retinopathy and nephropathy. Giving that ECFCs number and function are reduced in diabetic condition, how to obtain sufficient amount of ECFCs with desirable function for therapeutic is under investigation.

## 5. ECFC Therapy for Diabetic Microvascular Disease

### 5.1. Drugs

Till present, some of the antidiabetic drugs like metformin, thiazolidinediones, GLP-1 agonists, DPP-4 inhibitors, and insulin might increase EPCs number and improve EPCs function with increase of nitric oxide bioavailability [78–87]. Except for these antidiabetic drugs, lipid-lowering drugs, statins, improve EPCs number and function [88].  Table 1 provides an overview of these antidiabetic drugs in the aspects of their effects on EPCs number and function.

### 5.2. Cord- and Cord Blood-Derived ECFCs

For clinical practice, ECFCs can be obtained from long-term cultivation of mononuclear cells isolated from blood. Nevertheless, the quality and functionality of ECFCs could vary from one group to another, which could be due to the different passages and ECFCs purity they use and risk factors that the donors carry on [89].

Alternatively, induced pluripotent stem cells (iPSCs) generated from CD34^+^ cord blood cells have shown huge capability of differentiating into ECFCs. For therapeutic purposes, autologous iPSCs are more favored to avoid immune rejection. However, the experimental practice of iPSC generated from patients is always frustrating by growth arrest, uncontrolled differentiation, and incomplete function [90, 91]. Recent studies shed light on getting desired ECFCs. After iPSCs were obtained from healthy donors and patients with type-1 diabetes, the differentiation of iPSCs toward vascular cells was processed in an adherent, feeder-free differentiation protocol and further assembled in 3D engineered hyaluronic acid hydrogels for maturation. When injecting, the yielded endothelial progenitors were incorporated into vasculature [92]. In line with this study, Park et al. reported that vascular progenitors generated from human iPSCs derived from cord blood possessed greater capacity for homing and long-term incorporation into injured retinal vessels [2]. These studies establish promising strategies for applying iPSC-derived endothelial progenitors for stabilizing microvascular structure and inhibiting vessel leakage.

### 5.3. Genetically Modified ECFC

When dissecting the two main pathological machineries that affect retinopathy and nephropathy, applying ECFC with higher level of nitric oxide or Ang1 would be favorable for stabilized capillaries by reversing “uncoupled VEGF with nitric oxide,” balancing “Ang1/Ang2 competition” and “rendering Ang1/VEGF.” This idea awaits future evaluation.

## Figures and Tables

**Table 1:** Overview of the effect of antidiabetic drugs on EPCs number and function.

Author	Drug	Groups	Conclusion
Sorrentino et al., 2007	Thiazolidinedione (TZD)	T2DM patients T2DM: *n* = 30 Controls: *n* = 10	Rosiglitazone restored nitric oxide bioavailability and improved EPC function

Spigoni et al., 2012	Thiazolidinedione (TZD)	*In vitro *cultured ECFC isolated from subjects with impaired glucose tolerance: *n* = 14	Pioglitazone improved ECFC viability and capacity to form tubular-like structures

Humpert et al., 2008	Insulin	ECFC culture, *in vitro*	Insulin improved EPC function

Fadini et al., 2011	Insulin	T2DM patients: *n* = 42	Insulin increased EPC count

Maiorino et al., 2016	Insulin	T1DM patients insulin infusion: *n* = 41	Insulin infusion increased EPC number in T1DM patients

Liao et al., 2010	Metformin	Newly diagnosed T2DM: *n* = 46 Non-DM: *n* = 51	Metformin increased EPC number in the blood

Liu et al., 2011 [62]	GLP-1 agonists	EPC culture, *in vitro*	GLP-1 enhanced EPC proliferation and VEGF production in EPC

Gonçalves et al., 2012	DPP-4 inhibitors	Diabetic rats	Sitagliptin increased the number of CD34^+^ cells in the blood

Mohler III et al., 2009	Atorvastatin; ezetimibe	Diabetic swines	EPC number in the circulation was increased by atorvastatin

Chang et al., 2010	Adiponectin	Adiponectin deficient db/db mice	Adiponectin rescued EPC senescence

## Additional Points

Endothelial progenitors play a major role in the maintenance of endothelium integrity. Unfortunately, hyperglycemia induces metabolic and epigenetic changes in EPCs, leading to EPC apoptosis and reduced function. Reduced EPCs number and function jeopardize endothelial regeneration, resulting in hyperpermeability and inflammation in capillaries and then development of retinopathy and nephropathy. Providing the angiogenesis potential of incorporating into injured vessel and increased nitric oxide production upon stimulation, ECFCs might become promising and complimentary therapeutic targets for treating diabetic patients. iPSC-based ECFCs would be one of the main strategies for clinical therapeutic for diabetic microvascular abnormality. To gain intact endothelium and protect vessel from retinopathy and nephropathy, it might be advisable to administer ECFC in the early stage of diabetes for better efficacy.
